# Clay—Hand-Book of Obstetric Surgery

**Published:** 1874-11

**Authors:** 


THE COMPLETE HAND-BOOK OF OBSTETRIC SURGERY; or, Short Rules of Practice in every emergency, from the simplest to the most formidable operations connected with the science of obstetricy; with numerous illustrations. By Charles Clay, M.D., late Senior Surgeon and Lecturer on Midwifery, St. Mary's Hospital, Manchester, etc. From the third London edition. Philadelphia: Lindsay & Blakiston; 1874; 12mo; cloth; pp. 328. Price $2.25. Sold in Atlanta by J. J. & S. P. Richards.

In opening this handsome little volume, sent us by courtesy of the publishers, we have been led to promise ourselves a treat from the life-long experiences of so active and intelligent an observer as Dr. Charles Clay, of Manchester, most certainly is. Glancing over the comprehensive title-page, given above, we are raised to the tip-toe of expectancy, and inwardly exclaim—here we shall find intellectual pabulum, both short and sweet, terse and pithy, abounding in concentrated nutriment, both rich and rare. In closing our hasty sketch of its contents, however, we are forcibly reminded of the vanity of human expectations.

We are assured by the author in his preface, dated at Manchester, in May, 1874, that the volume has been carefully revised, and "it is hoped that in its present form it will prove a truthful guide to young practitioners," etc. Of this let us see.

In the chapter upon chloroform in labor, we are reminded that only partial anæsthesia is to be aimed at; we are to use a pocket-handkerchief, "into which pour a *drachm or more* of chloroform." We are admonished to "never commence chloroform in large doses." Let us modestly inquire what Dr. Clay would esteem a *large* dose of chloroform?

We turn to catheterism. "In applying the instrument, some recommend tracing downward in the median line from above, *over the prepeutium clitorides, clitoris*, (the italics are ours,) upper fissure of labia minora, and vestibule, when the index-finger falls into the circular depression (sic?) of the meatus urinarius." The author gives his own method as follows: "A small basin near the parts. The accoucher now takes the catheter in the *left hand*, previously smeared over with lard or spermaceti. He then passes his right hand beneath the clothing, under the thigh, and introduces the index-finger into the entrance of the vagina, and raises the finger until it gently presses on the under edge of the arch of the pubis, the palm of the finger being at this time upwards, and towards the symphysis pubis. The left hand, with the catheter, is now passed under the clothing, to the position of the right hand, and the instrument slid gently over the palm of the index-finger of the right hand, when, if properly managed, it enters at once into the meatus urinarius," etc. The latter direction is as imperfect and inelegant as the former is—in the American as well as in the English sense—"nasty."

The author claims the introduction of ergot into British obstetricy as far back as 1823. He recommends it to assist labor, and does not require that the os be dilated, only dilatable. He deprecates the pharmaceutical preparations of the drug, and insists upon the freshly-made decoction. The important topic of *erysipelas* is dispatched in a single sentence: "Whether in adults or infants, try painting: in the adult, with tinct. iodine; in infants, dilute it with half water."

The methods of treatment of vesico-vaginal fistula are *primitive* to a degree. We cannot better characterise the handling of the subject than by giving the author's—"Prognosis—not favorable; spontaneous cures have been effected when the injury has been at the juncture of the urethra and bladder; if posteriorly, and much loss of substance, the case is generally hopeless." We turn back instinctively, to see if the preface be not one of 1814, instead of 1874. The author speaks guardedly of the use of the forceps: "We ourselves have had an experience of from 13,000 to 14,000 cases, and certainly have not used the forceps 200 times, half of which number occurred in the practice of others, to whom we were called to render assistance;" and still we are presented on page 91 with an illustration of the method of applying the forceps to the *chest of the child!*

Dr. Clay speaks doubtingly of the cure of ruptured purineum: "These radical attempts may fail to cure; then compresses, and a spring-bandage the only remaining relief." Of the operation of ovariotomy, as recorded in this good year 1874, we are astonished at the claim: "In fact the number of cases I have diagnosed and operated upon exceeds the number of recorded cases from other parties, both native and foreign. I have now operated seventy-one times," etc. Surely our author has made his *revision* in an afternoon's nap!

The mortality of breach labors is stated to be one child in seven. The directions for treatment: "Announce a cross birth; prepare for infant asphyxia," etc., are both too tragical and too antiquated for 1874. The directions for prolapse of the cord are far behind the times, and so also of face labors. The author's ideas of specula are altogether elementary. Of sponge tents we are informed they "are made by soaking fine sponge in mucil. acac., and folding it round a stiletto, and then tied with a string; when dry it can be cut into any form."

Under the heading "ulceration of os or cervix," we find—"I have great success by using a long male glass syringe, very small in the bore, and from ten to twelve inches in length (made purposely); with this I dress the case by introducing the point within the os, and injecting the fluid into the cavity of the uterus. Different preparations are used, consisting of carbolic acid, iodine, tannin, etc., accompanied with constitutional treatment. As many cases of the ulcerated os and cervix are given up because they do not yield to treatment, the seat of disease being higher up in the cavity, it has therefore not been reached by the usual mode of application. The injection I find most effective is the following: ℞. carbolic acid, ℥ss; tinct. iodine, ℥ss; glycerin of tannin, ℥j. m. ft. inject. One teaspoonful drawn into the syringe will be sufficient for a dressing, and applied every other day. I use no speculum, but place the index-finger of the right hand on the os uteri, then slide the point of the syringe along the finger until it enters the os uteri."

We forbear further comment, and feel that we have ample justification for regarding our author, as exhibited in his recent work, as another Rip Van Winkle, untimely waked from his peaceful slumbers.

